# The Role of Diet and Gut Microbiota in Alzheimer’s Disease

**DOI:** 10.3390/nu16030412

**Published:** 2024-01-31

**Authors:** D. M. Sithara Dissanayaka, Vijay Jayasena, Stephanie R. Rainey-Smith, Ralph N. Martins, W. M. A. D. Binosha Fernando

**Affiliations:** 1 Centre of Excellence for Alzheimer’s Disease Research & Care, School of Medical and Health Sciences, Edith Cowan University, Joondalup, WA 6027, Australia; sitharad@our.ecu.edu.au (D.M.S.D.); stephanie.raineysmith@murdoch.edu.au (S.R.R.-S.); r.martins@ecu.edu.au (R.N.M.); 2 Alzheimer’s Research Australia, Ralph and Patricia Sarich Neuroscience Research Institute, Nedlands, WA 6009, Australia; 3 School of Science and Health, Western Sydney University, M15, Rm. G54, Locked Bag 1797, Penrith, NSW 2751, Australia; v.jayasena@westernsydney.edu.au; 4 Centre for Healthy Aging, Murdoch University, Murdoch, WA 6150, Australia; 5 Department of Biomedical Sciences, Faculty of Medicine, Health and Human Sciences, Macquarie University, Sydney, NSW 2109, Australia

**Keywords:** Alzheimer’s disease (AD), gut–brain axis, dietary components, gut microbiota

## Abstract

Alzheimer’s disease (AD), the most prevalent form of dementia, is characterized by the accumulation of amyloid-beta (Aβ) plaques and hyperphosphorylated tau tangles. Currently, Alzheimer’s disease (AD) impacts 50 million individuals, with projections anticipating an increase to 152 million by the year 2050. Despite the increasing global prevalence of AD, its underlying pathology remains poorly understood, posing challenges for early diagnosis and treatment. Recent research suggests a link between gut dysbiosis and the aggregation of Aβ, the development of tau proteins, and the occurrence of neuroinflammation and oxidative stress are associated with AD. However, investigations into the gut–brain axis (GBA) in the context of AD progression and pathology have yielded inconsistent findings. This review aims to enhance our understanding of microbial diversity at the species level and the role of these species in AD pathology. Additionally, this review addresses the influence of confounding elements, including diet, probiotics, and prebiotics, on AD throughout different stages (preclinical, mild cognitive impairment (MCI), and AD) of its progression.

## 1. Introduction

Unlike the typical aging process, Alzheimer’s disease (AD) is a progressive neurodegenerative condition characterized by a range of cognitive impairments affecting various aspects of daily life. These impairments impact memory, thinking, decision making, communication, problem solving, personality, and mobility [1,2]. In AD, the formation of amyloid-beta (Aβ) plaques and hyperphosphorylated tau neurofibrillary tangles (NFTs) leads to inflammation and a gradual decline in cognitive function [3]. Despite various hypotheses about the development of AD, its onset and progression remain unclear.

Recent evidence suggests that the gut microbiota–brain axis could offer insights into the early diagnosis and treatment of neurodegenerative disorders, including depression and AD [4,5,6]. Gut health is significantly influenced by microbiota, which is largely composed of diverse microorganisms and resides primarily in the gastrointestinal tract (GIT). The gut microbiota’s role in AD pathogenesis has been extensively explored, revealing that individuals with AD and mild cognitive impairment (MCI) exhibit a lower gut microbiota diversity index than healthy controls [7,8].

Additionally, studies indicate similarities in the gut microbiota of individuals with MCI and AD, offering potential insights into pre-dementia pathogenesis and the identification of at-risk individuals [9,10]. Moreover, numerous studies are pursuing the goal of understanding and mitigating changeable risk factors for AD pathology, such as lifestyle, different types of dietary patterns, and obesity. These external factors play a critical role in AD development [11]. Conversely, research has shown that a healthy diet may offer a non-pharmacotherapeutic approach to modulating AD neuropathological markers [12]. Therefore, researchers are studying several lifestyle and dietary patterns in order to determine which patterns are most effective in preventing AD, focusing primarily on the Mediterranean diet, DASH diet, MIND diet, and ketogenic diet [13].

Gut microbiota can be affected by several factors, including genetics, age, antibiotics, and diet. Hence, this review aims to enhance our understanding of gut microbiota function, the role of diet, and the connections of these factors to AD.

## 2. Alzheimer’s Disease

AD is a neurodegenerative disease that affects 50 million people worldwide. It is estimated that this number will reach 152 million by 2050 [14,15]. AD is the 6th leading cause of death among adults due to a decline in memory and cognitive functions [16]. Currently, in Australia, one in ten people over 65 have AD, and three in ten people over 85 have the disease [17]. In Australia, dementia is the second leading cause of death for all residents, and according to provisional data, it is expected to become the leading cause of death within the next few years. According to Austrian statistics, there are estimated to be almost 29,000 people suffering from young-onset dementia in 2024, and the number is expected to rise to over 41,000 individuals by 2054. It can include individuals in their 30s, 40s, and 50s [18]. A global estimate of the annual cost of AD and other forms of dementia is USD $605 billion, equivalent to 1% of the global gross domestic product [19]. It is predicted that by 2030, the costs associated with AD and dementia will more than double from US$1.3 trillion per year to $2.8 trillion dollars per year, according to the World Alzheimer Report 2023 [15,20]. There is no effective treatment for AD, which results in symptoms worsening as the condition progresses. AD has been recognized as a global public health priority by the World Health Organization (WHO) [21].

### 2.1. Pathology of Alzheimer’s Disease

The neuropathological hallmarks of AD are extracellular Aβ plaques and the formation of NFTs. The development of Aβ and NFTs results in the loss of synapses and neurons [22]. Aβ plaques develop initially in the basal, temporal, and orbitofrontal neocortex regions of the brain and eventually spread into the neocortex, hippocampus, amygdala, diencephalon, and basal ganglia [23]. There are several hypotheses that have been proposed to explain the mechanism of action of Aβ peptides and NFTs in neurodegeneration of AD [22]. These include the amyloid cascade hypothesis, the tau hyperphosphorylation hypothesis, and the oxidative stress hypothesis. In addition, oxidative stress, mitochondrial dysfunction, and neuroinflammation play crucial roles in neuropathological changes in the brain [24].

### 2.2. Amyloid-Beta Peptides

Aβ is a transmembrane protein that is produced by hydrolysing the amyloid precursor protein (APP) via the amyloidogenic pathway [25]. This process is initiated by beta-site APP cleaving enzyme 1 (Beta-Secretase 1 (BACE1)), which forms a large soluble protein and a 99-amino-acid C-terminal fragment (C99). The C99 fragment is further processed by a γ-secretase to produce Aβ in either its 40- or 42-amino acid form [26]. Aβ42 levels have been identified as being important in early events in AD pathogenesis, especially the ratio of Aβ42/Aβ40 [26]. Further, Aβ monomers aggregate into oligomers, protofibrils, and amyloid fibrils. Fibrils of amyloid are larger and insoluble, and they can form plaques, whereas oligomers of amyloid can travel throughout the brain [26,27].

### 2.3. Tau Proteins

An NFT consists of paired helical filaments and straight filaments, which contain an abnormally phosphorylated form of the microtubule-associated protein tau. Tau is mainly found in neuronal axons of the brain [28]. When tau is acetylated or truncated, it is unable to bind to microtubules, which promotes tau aggregation, mitochondrial dysfunction, and synaptic deficiencies [29].

### 2.4. Oxidative Stress

Oxidative stress plays a significant role in the development and progression of AD pathology. Oxidative stress occurs when there is an increase in free radicals, such as reactive oxygen species (ROS) and reactive nitrogen species [30]. Under stressful conditions, ROS formation increases within mitochondria and increases the risk of developing AD. In AD, oxidative stress promotes Aβ deposition and tau hyperphosphorylation, as well as subsequent loss of synapses and neurons [31].

## 3. The Gut–Brain Axis

The bidirectional communication between the enteric nervous system (ENS) and the central nervous system (CNS), known as the gut–brain axis (GBA), establishes a connection between the emotional and cognitive functions of the brain and peripheral intestinal functions [32,33]. Recent studies highlight the significant role of gut microbiota in GBA function, influencing the nervous, immune, and endocrine systems [34]. The microbiota in the gut produces various of neuroactive substances, such as neurotransmitters, short-chain fatty acids (SCFAs), and other bacterial metabolites, which influence neural activity and brain function [34,35,36,37,38]. These substances are generated through the fermentation of dietary fibre and other components (such as nitric oxide, ammonia, and ethanol) by gut bacteria [35,39].

However, gut dysbiosis has been linked to conditions such as anxiety, depression, autism spectrum disorders, and AD [40,41]. Consequently, the GBA has emerged as a potential target for therapeutic interventions aimed at enhancing brain health and treating neurological and psychiatric disorders. Enhancing brain health and treating brain-related disorders can be achieved by modifying the gut microbiota through various interventions, including dietary adjustments, prebiotics, probiotics, antibiotics, and faecal microbiota transplantation [42,43,44]. Further research is required to gain a more profound understanding of the complex interactions between the gut microbiota and the brain and to develop effective therapeutic strategies based on this knowledge.

## 4. Gut Microbiota

The human gut microbiota comprises bacteria, fungi, archaea, viruses, and protozoans existing in symbiotic relationships within the gastrointestinal tract [45]. In the human intestine, there are approximately 1000 species and 7000 strains of bacteria, with Firmicutes (such as *Lactobacillus*, *Clostridium*, and *Eubacterium*) and Bacteroidetes (including *Bacteroides*, and *Prevotella*) being the predominant phyla [33,45,46]. Recent studies on the human microbial flora emphasize the importance of maintaining a healthy intestinal microbiome, as there is a continual fluctuation in the structure, quantity, distribution, and biological characteristics of the endogenous intestinal flora.

An imbalance in intestinal flora is implicated in various diseases, including AD. Gut microbiota imbalance is closely associated with deficiencies in gut barrier function and intestinal permeability. A compromised gut barrier can lead to the release of microbial metabolites into the bloodstream. If the blood–brain barrier (BBB) experiences leakage, several proinflammatory cytokines can enter the central nervous system, triggering neuroinflammation by activating microglia and astrocytes [34,47].

## 5. Relationship between Gut Microbiota and AD

The microbiome’s involvement in AD pathogenesis has been observed in both animal and human studies. Specifically, research has identified associations between certain microbial organisms and the levels of cerebrospinal fluid (CSF) biomarkers related to AD. As an example, associations were noted between lower levels of cerebrospinal fluid (CSF) biomarkers, including the Aβ42/Aβ40 ratio, phosphorylated tau (p-tau), and the p-tau/Aβ42 ratio, and the presence of *Clostridiaceae* (SMB53) and *Erysipelotrichaceae* (cc115). Conversely, *Blautia* and *Bacteroides* spp. were associated with higher CSF biomarker levels [48]. Verhaar et al. (2022) introduced a machine learning model that identified *Lachnospiraceae* spp., *Lachnoclostridium edouard*, and *Blautia faecis* (Firmicutes) as the leading microbes in predicting the presence of tau, based on the area under the curve (AUC). This study also highlighted an association between gut microbiota composition and amyloid levels in the brain [49]. In five cross-sectional studies, associations were detected between the phylum Bacillota (*Lachnospiraceae*, *Ruminococcus torques*, *Roseburia hominis*, *Lachnoclostridium*, *Marvinbryantia* spp.) and the levels of Aβ and tau in both plasma and cerebrospinal fluid (CSF) [48,50,51,52,53]. Conversely, research identified higher levels of *Alistipes* spp. and *Odoribacter splanchicus* associated with increased amyloid in the CSF and decreased p-tau in the CSF [50]. Li et al. (2019) identified a negative correlation between amyloid burden and *Lactobacillus* abundance, as well as a positive correlation between *Akkermansia muciniphila* (phylum: Verrucomicrobiota) and medial temporal lobe atrophy [9]. Evidence from clinical and preclinical studies indicated that *A. muciniphila* plays a significant role in the development of depression, anxiety, Alzheimer’s disease, Parkinson’s disease, and other neuropsychiatric disorders [54]. However, some researchers discovered that *A. muciniphila* significantly reduced cognitive impairment in AD mouse models. It also improved the abundance of gut microbes that produce SCFAs and neurotransmitters. Additionally, they found that *A. muciniphila* reduced Aβ1–42 deposition in AD mice [55,56,57]. However, the underlying mechanism of *A. muciniphila*’s effect remains controversial.

Furthermore, Li et al.’s study detected associations between higher abundances of *Fusicatenibacter*, *Blautia*, and *Dorea* (family: *Lachnospiraceae*) and lower scores on the Mini-Mental State Examination (MMSE) among AD patients (18.1), individuals with mild cognitive impairment (MCI) (27.2), and normal controls (29.1). However, the presence of *Hungatella*, *Faecalibacterium*, and *Butyricicoccus* (family: *Clostridiaceae*) was associated with higher MMSE scores [9].

Both animal and human clinical research have reported alterations at the phylum level in the gut microbiota of AD patients and AD transgenic animal models, specifically in Firmicutes, Proteobacteria, and Bacteroidetes [58]. Although changes in *Actinobacteria* and *Verrucomicrobia* have been observed, these phyla are less prevalent in the gut of individuals with AD [58,59]. Cattaneo et al. (2017) demonstrated that individuals with amyloidosis exhibit distinct gut microbiota compositions compared to those without brain amyloidosis [48]. This study also revealed increased levels of proinflammatory cytokines, such as interleukin (IL)-6, CXCL2, NLRP3, and IL1β, in amyloid-positive patients compared to the anti-inflammatory cytokine IL-10. Proinflammatory cytokines were positively correlated with *Escherichia*/*Shigella* and negatively correlated with *Eubacterium* [48]. Additionally, Vogt et al. (2017) observed a reduction in gut microbiome bacterial diversity in AD patients compared to healthy age- and sex-matched control subjects. This was determined by sequencing 16S rRNA amplicons from faeces isolated from AD patients with dementia. Furthermore, they noted a decrease in Firmicutes and Bifidobacterium along with an increase in Bacteroidetes in AD patients compared to healthy controls (HCs) [50]. Microbiome studies conducted in China have also revealed differences in gut microbiota composition between AD patients and HCs [9,51,53,60]. Studies of human cohorts have also revealed links between specific gut bacteria and AD based on differentially abundant taxa (Table 1).

Li et al. (2019) study revealed higher prevalence of Bacillota (*Blautia*, *Dorea*), Firmicutes (*Lactobacillus*, *Streptococcus*), Verrucomicrobiota (*Akkermansia*), Actinobacteria (*Bifidobacterium*), and Pseudomonadota (*Acinetobacter*) in individuals with AD compared to healthy controls (HCs) [9]. Additionally, five cross-sectional studies identified *Odoribacter splanchnicus*, *Bacteroides*, *Prevotella*, and *Alistipes* spp. as highly abundant in AD, while *Faecalibacterium prausnitzii*, *Eubacterium*, *Anaerostipes*, *Ruminococcus*, and *Roseburia* spp. showed lower abundances in AD patients than in HCs [50,51,52,53,61]. Other reports also observed increased relative abundance of Actinobacteria and Bacilli, along with decreased relative abundance of *Negativicutes* and *Bacteroidia* in the AD group [48,50,51,52].

Although these findings collectively suggest alterations in gut microbiota composition in AD patients, it is essential to note that these studies primarily establish correlations, and there is a lack of uniformity in the outcomes regarding the bacterial phyla altered in AD patients.

**Table 1:** Alterations in microbial diversity associated with AD from human studies.

Sequencing Methods	Sample Size	Year	Results		Reference
			Decreased Microbiota Diversity in AD	Increased Microbiota Diversity in AD	
16S rRNA amplicon sequencing for faecal samples	25 AD 25 HCs	2017	Phyla: Firmicutes, Actinobacteria Genera: *Bifidobacterium*, *SMB53*, *Dialister*, *Clostridium*, *Turicibacter*, *Adlercreutzia*, *cc115*	Phylum: Bacteroidetes Genera: *Blautia*, *Bacteroides*, *Alistipes*, *Phascolarctobacterium*, *Bilophila*, *Gemella*	[50]
qPCR for faecal samples	40 amyloid-positive 33 amyloid-negative 10 HCs	2017	Amyloid-positive group showed lower abundance of E. rectale than other groups	Amyloid-positive group showed higher abundance of *Escherichia/Shigella* than other groups	[48]
16S rRNA amplicon sequencing for faecal samples	43 AD 43 HCs	2018	Phylum: Actinobacteria Classes: Negativicutes, Bacteroidia Orders: Bacteroidales, Selenomonadales Families: Lanchnospiraceae, Bacteroidaceae, Veillonellaceae Genera: *Lachnoclostridium*	Phylum: Bacteroidetes Classes: Actinobacteria, Bacilli Order: Lactobacillales Families: Ruminococcaceae, Enterococcaceae, Lactobacillaceae Genera: *Bacteroides*, *Ruminococcus*, *Subdoligranulum*	[51]
NextSeq500/Metagenomic analysis for faecal samples	24 AD 33 with other dementia types 51 HCs	2019	Genus: *Lachnoclostridium*	Genera: *Bacteroides*, *Alistipes*, *Odoribacter*, *Barnesiella*	[52]
16S rRNA amplicon sequencing for faecal and blood samples	30 AD 30 MCI 30 HCs	2019	Genera: Alistipes, Bacteroides, Parabacteroides, Sutterella, Paraprevotella	Genera: Dorea, Lactobacillus, Streptococcus, Bifidobacterium, Blautia, Escherichia	[9]
16S rRNA amplicon sequencing for faecal samples	33 AD 32 amnestic MCI 32 HCs	2019	Phylum: Firmicutes	Phylum: Proteobacteria Orders: Gammaproteobacteria, Enterobacteriales Family: Enterobacteriaceae	[53]
16S rRNA amplicon sequencing for faecal samples	100 AD 71 HCs	2021	Genus: butyrate-producing *Faecalibacterium*	Genus: lactate-producing *Bifidobacterium*	[62]
16S rRNA amplicon sequencing	20 MCI 22 HCs	2021	Genus: *Bacteroides* Families: Veillonellaceae, Ruminococcaceae	Genera: *Blautia*, *Bacteroide* Family: Lachnospiraceae	[63]
16S rRNA amplicon sequencing for faecal samples	11 MCI 11 AD 34 HCs	2022	Phylum: Firmicutes Genera: *Bilophila*, *Faecalibacterium* Classes: Clostridia, Deltaproteobacteria Orders: Clostridiales, Desulfovibrionales Families: Lachnospiraceae, Desulfovibrionaceae, Ruminococcaceae	Phylum: Bacteroidetes Class: Bacteroidia Order: Bacteroidal	[64]
16S rRNA amplicon sequencing for faecal samples	27 MCI 47 AD 51 HC	2022	Genera: *Roseburia*, *Lactobacillus*, *Fusicatenibacter*	Genera: *Prevotella*, *Bacteroides*	[65]

AD, Alzheimer’s disease; MCI, mild cognitive impairment; HC, healthy control.

## 6. Diet, Alzheimer’s Disease, and Microorganisms

**Figure 1:**
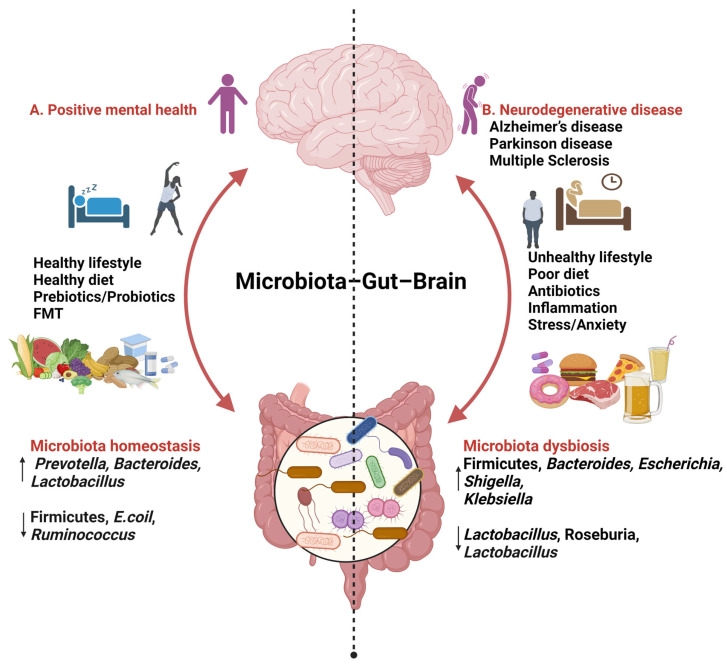
**Findings relating to gut–brain–microbiome interactions** (created with BioRender.com, 24 January 2024). ↑ increase ↓ decrease.

The impact of diet on health can be either beneficial or detrimental. There is a possibility that diet plays a role in influencing microbial communities [66,67]. Various factors such as dietary patterns, microbiome-specific interventions, and the consumption of natural supplements have the potential to significantly modify the composition of the microbiota. This alteration, in turn, affects the gut–brain axis (GBA), potentially leading to the alleviation of AD-related pathology [68,69] (Figure 1).

A. Consuming a diet rich in fats (MUFAs, PUFAs), carbohydrates (fibre), and proteins, along with incorporating probiotics, prebiotics, and engaging in daily activities such as good sleep and exercise, has been associated with improved mental health. This dietary pattern is linked to an increase in beneficial microbial species such as *Prevotella*, *Bacteroidetes*, and *Lactobacillus*, while concurrently reducing levels of Firmicutes, *Escherichia coli*, and *Ruminococcus*.

B. An unhealthy lifestyle marked by stress; anxiety; and the consumption of high-fat, high-sugar, and processed foods has been associated with an increase in *Firmicutes*, *Bacteroides*, *Escherichia*, *Shigella*, and *Klebsiella*, while simultaneously decreasing levels of *Lactobacillus*, *Roseburia*, and *Bacteroides*.

### 6.1. Dietary Protein and Gut Microbiota

Protein stands as an essential macronutrient required by the human body. The quantity and source of protein intake, whether animal- or plant-based, can impact overall health and brain function [70]. The prolonged consumption of protein may influence the risk of cognitive decline, with higher protein intake being associated with a lower level of subjective cognitive impairment [71]. According to Fernando et al. (2018), a diet rich in protein may have a protective effect against brain amyloid-beta (Aβ) burden, especially before the onset of objective memory decline in older adults [72]. During the digestive process, unabsorbed dietary protein undergoes fermentation by proteolytic bacteria, resulting in beneficial end products that influence both host function and the composition of the microbiota [73]. Protein-rich diets have been linked to a reduction in anti-inflammatory bacteria, such as *Bifidobacterium adolescentis*, in the intestine, along with an increase in proinflammatory bacteria such as *Bacteroides* and *Clostridium* spp. [74]. Furthermore, studies have indicated a correlation between the intake of animal-derived proteins and a higher prevalence of AD due to the production of neurotoxic end products [75]. Conversely, substituting plant-derived proteins has been associated with reduced dementia-related mortality and improved brain health. Additionally, animal proteins may contribute to inflammation by promoting the growth of anaerobic bacteria such as *Bacteroides*, *Alistipes*, and *Bilophila* [76,77,78]. In contrast, plant-based proteins stimulate the growth of probiotic microorganisms such as *Bifidobacterium* and *Lactobacillus* while reducing the growth of pathogenic taxa such as *Bacteroides fragilis* and *Clostridium perfringens* [76,79,80,81]. Studies have shown a reduction in Roseburia and Eubacterium rectale in the intestinal microflora and a decrease in butyrate in the faecal matter of individuals following a high-protein or low-carbohydrate diet.

### 6.2. Dietary Fibre and Gut Microbiota

In general, carbohydrates can be categorized into simple sugars (monosaccharides, disaccharides) and complex sugars (starch, fibre). Dietary fibre serves as a beneficial reservoir of “microbiota-accessible carbohydrates” (MACs), enabling microbes to provide the host with both energy and carbon. Additionally, fibre has the ability to alter the flora of the intestinal tract. Fibres are therefore recognized as prebiotics, and increased intake of simple sugars is associated with an elevated risk of AD, whereas higher fibre intake is linked to a reduced risk of AD [79,82]. The consumption of soluble fibre promotes the production of short-chain fatty acids (SCFAs) by gut bacteria [83,84]. Evidence suggests that soluble fibre intake reduces propionate formation, enhances butyrate production, diminishes the activation of astrocytes, and improves cognitive function in the APP/PS1 mouse model of AD; these effects are attributed to gut microbiota dysbiosis [85].

A study indicated that individuals with a high fibre intake exhibited an increased prevalence of probiotic bacteria, including *Lactobacillus*, *Bifidobacterium*, and *Roseburia*, consequently reducing the Firmicutes:Bacteroidetes ratio [78,80].

### 6.3. Dietary Fat and Gut Microbiota

Dietary fats come in two main types, namely, saturated and unsaturated; the consumption of fats such as free fatty acids (FFAs), monounsaturated fats (MUFAs), and polyunsaturated fats (PUFAs) can influence the functions of numerous beneficial microorganisms such as *Prevotella*, *Bifidobacterium* [78]. MUFAs and PUFAs are associated with positive effects, including enhanced brain function and the prevention of neurodegenerative diseases [86,87,88]. A high intake of saturated fats and trans fats is associated with an increase in proinflammatory bacteria, while a high intake of MUFAs and PUFAs enhances the production of short-chain fatty acid (SCFA)-producing bacteria.

Studies have shown that saturated fats reduce anti-inflammatory bacteria (*Lactobacillus intestinalis*) and increase proinflammatory bacteria (*Clostridial*, *Bacteroides*, *Bilophila*, and *Enterobacteriaceae*) in mouse models [89]. Studies related to humans showed that saturated fat consumption led to an increase in the phylum Actinobacteria, while the phylum Firmicutes decreased in human studies. Additionally, the consumption of fish oil, rich in omega-3 PUFAs, increased the abundance of beneficial microbes, including *Bifidobacterium*, *Adlercreutzia*, *Lactobacillus*, *Streptococcus*, and *Akkermansia muciniphila* in transgenic mouse models [90]. In human studies, omega-3 PUFAs were shown to decrease the Firmicutes:Bacteroidetes ratio and increase the abundance of SCFA-producing bacterial genera, such as *Bifidobacterium*, *Lachnospiraceae*, and *Roseburia* [91,92,93]. High MUFA intake leads to increased levels of bacteria such as *Parabacteroides*, *Roseburia*, and *Oscillospira*, while decreasing proinflammatory bacteria such as *Prevotella* [94].

### 6.4. Polyphenols

Polyphenols are micronutrients with antioxidant properties that naturally occur in plants and plant-based foods [95]. The majority of polyphenols can be found in fruits and vegetables such as grapes, blackcurrants, cocoa, black and green olives, oranges, apples, almonds, flax seeds, pomegranates, red onions, and tomatoes. It is also possible to find polyphenols in coffee, green tea, and wine [96,97]. Polyphenols have been shown to affect the composition and diversity of intestinal microbiota. A diet rich in polyphenols promotes the growth of beneficial microbes, such as *Bifidobacterium*, and *Lactobacillus*, and lowers levels of pathogens, including *Staphylococcus aureus*, *Salmonella typhimurium*, and *Clostridium* spp. [98,99]. In addition to their anti-inflammatory properties, polyphenols and their metabolites have been shown to prevent cognitive decline. It has also been found that dietary polyphenols may prevent neurodegenerative conditions through the AGEs-RAGE axis, as well as by regulating the microbiota–gut–brain axis [100].

### 6.5. Dietary Patterns and Gut Microbiota

**Figure 2:**
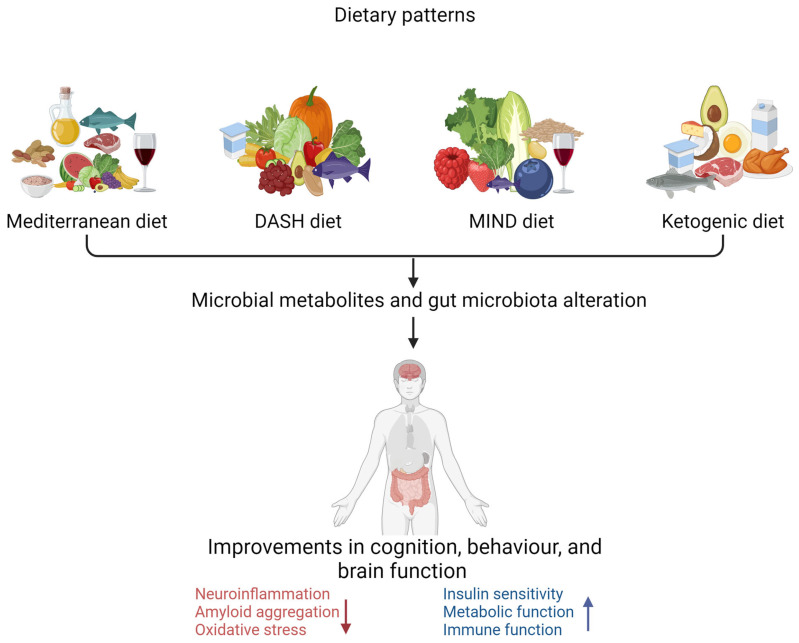
**Dietary pattern interventions to delay the progression of Alzheimer’s disease** (created with BioRender.com). Dietary patterns such as the Mediterranean diet, DASH diet (Dietary Approaches to Stop Hypertension), MIND diet (Mediterranean–DASH Intervention for Neurodegenerative Delay), and ketogenic diet are associated with improved cognitive, behavioural, and brain function. ↑ increase ↓ decrease.

A growing body of evidence indicates that alteration of gut microbiota due to diets rich in vegetables, legumes, grains, nuts, and fish, with a preference for plant-based foods over animal products, holds the potential to prevent the intestinal inflammatory processes that underlie many chronic diseases including AD [101,102]. The most studied dietary patterns are the Mediterranean diet (MeD), Dietary Approaches to Stop Hypertension (DASH), the Mediterranean–DASH Intervention for Neurodegenerative Delay (MIND), and the ketogenic diet (KD) in related to AD in the elderly population [68,103,104] (Figure 2).

There are key differences among the Mediterranean, MIND, DASH, and ketogenic diets, as illustrated in Table 2, regarding the types of foods consumed in each dietary pattern [105]. All these dietary patterns have been associated with neuroprotective properties. The Mediterranean diet is associated with a reduced risk of cognitive decline in populations that consume it [105,106]. Consumption of the DASH diet has also been associated with improved cognitive function and a lowered risk of AD [107,108]. Evidence indicates that the MIND diet reduces cognitive impairment risk [109]. There is evidence that a ketogenic diet reduces or delays cognitive impairment in older individuals through different pathophysiological mechanisms [110,111].

Several studies have been conducted to examine the relationship among the MeD, gut bacteria, and AD. The MeD consists of a diet rich in vegetables, fruits, nuts, whole grains, and olive oil, with moderate consumption of fish, poultry, and red wine, as well as polyphenols, fibre, and carbohydrates with a low glycaemic index. Therefore, the MeD promotes the growth of saccharolytic microbial species (Bacteroidetes, Firmicutes, and Actinobacteria) as well as the release of beneficial metabolites [115,116,117]. Consumption of the MeD has been shown to result in elevated levels of gut bacteria producing SCFAs, such as *Bifidobacterium*, *Roseburia*, and *Lactobacillus*, and reduced levels of proinflammatory bacteria such as *Prevotella* and *Clostridium* [97,118,119,120]. Additionally, the MeD has been linked to reduced human systemic inflammation through the promotion of beneficial fibre-degrading bacteria such as *Cellulosilyticus*, *Faecalibacterium prausnitzii*, and *Eubacterium eligens* [121]. Based on a meta-analysis, MeD diet compliance is also associated with reduced MCI and AD risk. This study included 34,168 participants, demonstrating a reduction of 17% in the risk of MCI and a reduction of 40% in the risk of AD. In addition, 612 non-frail or pre-frail individuals across five European countries were examined over a 12-month period; the findings showed that inflammation was reduced and that cognitive function was improved [122,123]. Mediterranean-diet followers had a 20% lower risk of dementia, according to a study conducted in 16,160 elderly participants in the EPIC-Spain Dementia Cohort [124]. Based on a systematic review, the Mediterranean diet has been shown to have beneficial effects on the cognitive function of the aging population after 10 weeks of adherence [125,126].

The ketogenic diet (KD), characterized by high fat (75%) and protein (20%) intake, with minimal carbohydrates (5%), aims to induce a state of ketosis [127]. Studies on individuals with MCI or AD show significant improvements in cognitive function with KD consumption, along with alterations in the microbiota, affecting species such as *Akkermansia* and *Parabacteroides* [128,129]. However, research on humans suggests that KD consumption reduces beneficial microbes, including *Bifidobacteria*, *Dialister*, *E. rectale*, *Bacteroides*, and *Roseburia*, while increasing proinflammatory bacteria such as *E. Coli* and *Desulfovibrio* spp. [130,131,132,133].

A study in MCI individuals on a high-fat modified Mediterranean KD (MMKD) observed changes in GABA-producing bacteria and GABA levels, emphasizing the need for caution in prolonged fasting due to potential risks of toxic levels and ketoacidosis in older individuals [128]. Moreover, high adherence to the DASH and MIND diets has been associated with reduced AD risk. Moreover, these dietary patterns contain nutrients with antioxidant and anti-inflammatory properties, contributing to the suppression of Aβ deposition [103,122].

## 7. Prebiotics, Probiotics, and Alzheimer’s Disease

Probiotics are living bacteria that promote the health of the host, while prebiotics are fibre substances degraded by gut microbiota [43,134]. The use of probiotics, such as lactic acid bacteria and *Bifidobacterium*, to reduce neuroinflammation has attracted attention [135,136]. However, there is still limited research on the therapeutic effects of probiotics and prebiotics in AD.

### 7.1. Prebiotics and Alzheimer’s Disease

Prebiotics are substrates selectively metabolized by host microorganisms to generate health benefits [137]. Recent studies in both animals and humans have investigated the impact of prebiotics on mental health. As an illustration, Liu et al. (2021) treated 5XFAD mice with the prebiotic mannan oligosaccharide, noting decreases in cognitive deficits, amyloid plaques, oxidative stress, and microglial activation, alongside modifications in the gut microbiome [138]. Additionally, Chen et al. (2017) noted that the prebiotic R13 tropomyosin receptor kinase B (TrkB) inhibited the proinflammatory pathway in the gut, leading to reduced amyloidogenesis and oxidative stress [28]. The prebiotic sodium oligomannate (GV-971) is utilized to enhance cognitive function and treat mild to moderate AD. Evidence suggests that GV-971 can reverse cognitive impairment; rectify gut dysbiosis; suppress neuroinflammation; and permeate the blood–brain barrier to directly bind to Aβ, inhibiting Aβ fibril formation [139].

### 7.2. Probiotics and Alzheimer’s Disease

The microbiota associated with probiotics can enhance cognitive function and play a positive role in preventing memory loss in Alzheimer’s disease (AD) [140,141]. *Lactobacillus* and *Bifidobacterium* are among the most commonly utilized probiotic genera [43]. Eight weeks of consumption of *Bifidobacterium breve*, *Bifidobacterium longum*, and *Bifidobacterium infantis* resulted in altered intestinal microbiome composition and increased levels of SCFAs in serum (acetate), brain (lactate and acetate), and serum (acetate and lactate). Notably, the proliferative marker Aβ and glial fibrillary acid protein did not exhibit significant changes [142,143].

An additional study demonstrated that combining probiotics with vitamin formulations resulted in reduced Aβ levels and improved cognitive performance in transgenic mouse models [144]. The same research group observed that when *Bifidobacterium lactis* Probio-M8 was administered for 45 days, it resulted in a reduced number of Aβ plaques, alterations in gut microbiota composition, and improved cognitive performance [145]. As per Akbari et al. (2016), probiotic milk containing *Lactobacillus acidophilus*, *Lactobacillus casei*, *Bifidobacterium bifidum*, and *Lactobacillus fermentum* is associated with significantly improved Mini-Mental State Examination (MMSE) scores, reduced plasma malondialdehyde (MDA), and decreased plasma C-reactive protein (CRP) [146]. When MCI patients were treated with *Bifidobacterium breve* A1 for 16 weeks, the findings showed a significant improvement compared to the placebo group in neuropsychological assessments, including the Repeatable Battery for the Assessment of Neuropsychological Status (RBANS) and the Japanese version of the MCI Screen (JMCIS) [147].

These studies concluded that probiotics, prebiotics, synbiotics (combinations of probiotics and prebiotics), and postbiotics (functional bioactive compounds such as SCFAs), could modify AD-related neuropathology and disease progression effectively, presenting a novel therapeutic approach [12,147].

## 8. Conclusions

Gut dysbiosis assumes a pivotal role in the pathology of AD, offering a non-invasive diagnostic and potential treatment avenue. The intricate interplay between gut microbiota and AD pathogenesis involves abnormalities in Aβ, tau phosphorylation, neuroinflammation, dysregulation of neurotransmitters, and oxidative stress. While various studies have identified functional bacteria linked to AD pathology and altered brain function, conclusive results remain elusive. Factors contributing to this ambiguity include a focus on genus-level associations without delving into species-level specifics and a lack of consideration for dietary changes. Ongoing research endeavours are dedicated to unravelling these mechanisms, promising valuable insights into the nuanced contributions of gut microbiota and dietary influences on cognition, dementia, and AD.

## Figures and Tables

**Table 2:** Components of the Mediterranean, MIND, DASH, and ketogenic diets.

	Mediterranean Diet [105,112]	MIND Diet [105,113]	DASH Diet [114]	Ketogenic Diet [112]
Moderate to high consumption	Whole grains, vegetables, fruits, olive oil, olives, nuts, seeds, potatoes, legumes, low-fat dairy, red wine, eggs, poultry, fish/seafood	Whole grains, beans, nuts, green leafy vegetables, berries, olive oil, poultry, fish, red wine	Grains, legumes, fruits, vegetables, nuts, seeds, poultry, fish, low-fat dairy	Meat, fish/seafood, high-fat dairy
Low consumption	Red meat, sweets, salt	Red meat, sweets	Red meat, sweets	
Restricted	-	-	-	Legumes, wines, beer, flour products, starch-rich vegetables, whole/refined grains, fruit juices

## Data Availability

Not applicable.
